# Cloning and Expression of a Novel Leucine Dehydrogenase: Characterization and L-*tert*-Leucine Production

**DOI:** 10.3389/fbioe.2020.00186

**Published:** 2020-03-31

**Authors:** Wei Luo, Jing Zhu, Yuzheng Zhao, Huili Zhang, Xue Yang, Yuantao Liu, Zhiming Rao, Xiaobin Yu

**Affiliations:** ^1^The Key Laboratory of Industrial Biotechnology, Ministry of Education, School of Biotechnology, Jiangnan University, Wuxi, China; ^2^State Key Laboratory of Bioreactor Engineering, East China University of Science and Technology, Shanghai, China; ^3^College of Life Sciences, University of Shihezi, Shihezi, China; ^4^Hulunbeier Northest Fufeng Biotechnologies Co., Ltd., Zhalantun, China

**Keywords:** leucine dehydrogenase, trimethylpyruvic acid, L-*tert*-leucine, reductive amination, amino acid

## Abstract

Among many genes encoding for amino acid dehydrogenase, a novel leucine dehydrogenase gene from *Exiguobacterium sibiricum* (*Esi*LeuDH) was isolated by using genome mining strategy. *Esi*LeuDH was overexpressed in *Escherichia coli* BL21 (DE3), followed by purification and characterization. The high thermostability of the enzyme confers its half-life up to 14.7 h at 50°C. Furthermore, the substrate specificity shows a broad spectrum, including many L-amino acids and aliphatic α-keto acids, especially some aryl α-keto acids. This enzyme coupled with recombinant formate dehydrogenase (FDH) was used to catalyze trimethylpyruvic acid (TMP) through reductive amination to generate enantiopure L-*tert*-leucine (L-Tle). In order to overcome the substrate inhibition effect, a fed-batch feeding strategy was adopted to transform up to 0.8 M of TMP to L-Tle, with an average conversion rate of 81% and L-Tle concentration of 65.6 g⋅L^–1^. This study provides a highly efficient biocatalyst for the synthesis of L-Tle and lays the foundation for large-scale production and application of chiral non-natural amino acids.

## Introduction

With the continuous development of the synthesis and analytical technologies (Bommarius et al., 1995; Taylor et al., 1998; Miyoshi et al., 2014), a growing number of unnatural amino acids are produced and used to synthesize antitumor and physiologically active peptides. Among these products with medicinal value, L-*tert*-Leucine (L-Tle) is an important non-natural amino acid, which is often used as a template to induce asymmetric synthesis and the product has high stereoselectivity (Lehr et al., 1996; Hong et al., 2010). This is ascribed to its *tert*-butyl side chain, which has a large steric hindrance and strong hydrophobic interaction because of the special structure of L-Tle. It is also widely used as a pharmaceutical intermediate in the treatment of cancer and AIDS and the production of biological inhibitors and peptide drugs (Bommarius et al., 1998; Hong et al., 2010).

**FIGURE 1:**
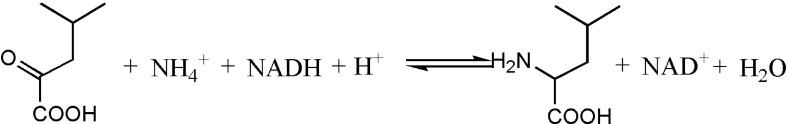
LeuDH catalyzes the synthesis of corresponding amino acids from ketoacid.

**FIGURE 2:**
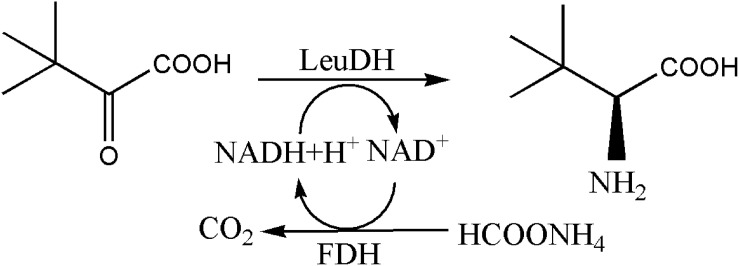
Synthesis of (S)-Tle by LeuDH coupling with FDH.

The synthesis method of L-Tle includes chemical and biological methods. Compared with traditional chemical synthesis (Pracejus, 1964; Steglich et al., 1971; Jaeger and Broadhurst, 1979; Kunz et al., 1989; Corey and Link, 1992), the enzymatic reduction of chiral keto acid to synthesize the corresponding unnatural amino acid has the advantages of high optical purity, mild reaction conditions, high conversion rate, and environmental friendliness (Kragl and Dwars, 2001; Ghislieri and Turner, 2014; Cheng et al., 2016). As the trigger of the enzyme reaction for the biosynthesis of L-Tle, Leucine dehydrogenase (LeuDH, EC 1.4.1.9) is a NAD^+^-dependent oxidoreductase that catalyzes the formation of chiral unnatural amino acids by chiral keto acids (Xu et al., 2017), as shown in Figure 1. Sanwal et al. (1961) first cloned and expressed LeuDH in *Bacillus cereus*, followed by the discovery in *B. sphaerius*, *B. stearothermophlius*, *Clostridium thermoaceticum*, and so on (Ohshima et al., 1978, 1985a,b; Shimoi et al., 2006), providing more ways for the biosynthesis of some chiral unnatural amino acids. In the redox reaction, the participation of the coenzyme Nicotinamide adenine dinucleotide (NADH) is required, so the yield of the α-amino acid can be increased if the coenzyme can be recycled (Kragl et al., 1996). Wichmann et al. (1981) have used formate dehydrogenase (FDH) to oxidize HCOONH_4_, efficiently providing reductive power for NADH regeneration. The reaction process shown in Figure 2 indicates that volatilization of the co-product CO_2_ promotes NADH formation and production of (S)-*tert*-leucine, leading to a yield of 85% and an *ee* value of 99%. Liu et al. (2014) constructed a co-expressing strain of LeuDH and FDH and explored the reaction conditions of catalystic synthesis of *tert*-leucine from trimethylpyruvic acid (TMP).

In this study, nine LeuDH genes were analyzed and singled out *via* genome mining method. Among them, *Esi*LeuDH from *Exiguobacterium sibiricum* exhibited higher activity in the reductive amination of TMP. The enzymatic properties of recombined LeuDH were characterized systematically, including the optimum temperature, optimum pH, kinetic parameters, and substrate specificity of the enzyme. Then, the crude extracts of *Esi*LeuDH were coupled with the recombinant FDH derived from *Lodderomyces elongisporus* in a reaction system for yielding of enantiopure L-Tle by reductive amination of TMP. The reaction parameters were optimized under batch mode prior to substrate fed-batch. Amino acid dehydrogenase has a promising prospect for the development of reduced keto acids, laying a foundation for large-scale production and application of chiral non-natural amino acid industry.

## Materials and Methods

### Microorganisms and Materials

The microbial strains *Lysinibacillus sphaericus* and *Stenotrophomonas maltophilia* were purchased from China General Microbiological Culture Collection Center. The *Bacillus sphaericus*, *B. subtilis*, *B. cereus*, and other microbes were purchased from Microbiological Culture Collection Center of Jiangnan University. *Escherichia coli* DH5α and BL21 (DE3) were cultured and preserved in our laboratory. TMP and L-Tle were acquired from West Asia Chemical Industry Co., Ltd. (Shandong, China). All enzymes used in this study were purchased from TaKaRa Co., Ltd. (Dalian, China). All other reagents were gained from China’s local market.

### Cloning and Expression of Leucine Dehydrogenase in *Escherichia coli*

Genomic DNAs were extracted from nine abovementioned strains by using the DNA extraction kits from Tian Gen (Shanghai, China), which were used as the templates for the cloning of LeuDH gene guided by oligonucleotide primers with restriction sites. The amplified DNA fragments were purified, digested, and inserted into pET28a(+), and then the recombinant plasmids were transformed into *E. coli* BL21 (DE3). The constructed recombinant cells were cultivated in Luria-Bertani (LB) Broth medium with the supplementation of kanamycin (final concentration of 50 μg⋅ml^–1^) at 37°C until the optical density (OD_600_) reaching 0.6–0.8. Then, temperature was immediately reduced to 25°C and 0.3 mM isopropyl β-D-1-thiogalactopyranoside (IPTG) was simultaneously added. A 15-h duration was needed for the induction of enzyme expression prior to cell collection.

### Purification of Recombinant Leucine Dehydrogenase

In order to remove impurities and harvest cells, the cultures were centrifuged at 9,000 g for 10 min and the supernatants were removed. Bacterial sediments were washed twice with saline solution and then resuspended in phosphate buffer saline (PBS) buffer (pH 8.5) for enzyme extraction. The cell suspensions were set in an ice bath to keep low temperature and treated by ultra-sonication to disrupt cells. Cells were crushed by two rounds of ultrasound treatment and centrifuged at 12,000 g for 20 min at 4°C. Enzyme purification was conducted on a Ni–NTA column pre-equilibrated with buffer (0.5 mol⋅L^–1^ NaCl, 20 mmol⋅L^–1^ sodium phosphate, 10 mmol⋅L^–1^ imidazole, pH 7.4), wherein the supernatant was loaded onto Ni-NTA agarose and then eluted by competition with imidazole. Gradient elution was carried out by imidazole solution at 1.0 ml⋅min^–1^ with an increasing concentration from 10 to 550 mM. The collected fractions were analyzed by sodium dodecyl sulfate polyacrylamide gel electrophoresis electrophoresis (SDS-PAGE) to determine the protein purities (Laemmli, 1970). Subsequently, all eluted fractions containing recombinant enzyme were gathered together and desalinated by dialysis treatment under buffer condition (20 mmol⋅L^–1^ NH_4_Cl-NH_3_⋅H_2_O, pH 8.5).

### Enzyme and Protein Assays

Enzyme assays regarding their activities were carried out on a spectrophotometer by detecting the change of NADH absorbance (ε = 6,220 M^–1^cm^–1^) at 340 nm for both reactions of reductive amination and oxidative deamination. The substrate mixture (1.5 ml) for enzymatic activity analyses contained 5 mM α-keto acid (or amino acid) and 0.2 mM NADH (or NAD^+^) in the presence of 1 M NH_4_Cl-NH_3_⋅H_2_O buffer (pH 8.5) (or 100 mM glycine–NaOH buffer, pH 9.5), wherein the difference of substrates mainly determines the activities of enzymes for reductive amination and oxidative deamination. One unit of enzymatic activity was defined as the amount of enzyme required to catalyze the oxidation of 1 μM NADH or the reduction of 1 μM NAD^+^ per minute at 30°C.

The purified enzyme was analyzed by the Bradford method to determine the protein concentration using bovine serum albumin as standard (Bradford, 1976). Specific activities were determined according to the calculation of enzyme activities divided by enzyme protein contents.

### Enzyme Stability Assay

Enzyme stability assays of LeuDH including thermostability and pH stability were determined by comparison of the initial and residual activities of LeuDH under different treatment conditions. For the thermostability assay, the purified enzyme was treated at 4, 30, 40, or 50°C for a certain period in the presence of NH_4_Cl-NH_3_⋅H_2_O buffer (pH 8.5). While for the pH stability assay, LeuDH was incubated in NH_4_Cl-NH_3_⋅H_2_O buffer with pH from 7.0 to 11.0 at 4°C for 24 h.

### Kinetic Analysis

The kinetic parameters of the enzyme, including the Michaelis–Menten constant (*K*_m_) and the maximal reaction rate (*V*_max_), were calculated from Lineweaver–Burk plots. Meanwhile, the *k*_cat_ value and *k*_cat_/*K*_m_ were calculated based on the molecular weight of the enzyme. Considering that it is a biochemical reaction involving double substrates whether for reductive amination or oxidative deamination, only the substrate to be determined was changed in content. To determine the kinetic parameters of the purified LeuDH for L-leucine, activities were measured under varied concentrations of L-leucine from 0.25 to 20 mM, while the concentrations of NAD^+^ were fixed at 1 mM. Similarly, to determine those kinetic parameters for TMP, TMP was added at a final concentrations from 2 to 50 mM to each reaction mixture with a fixed concentration of NADH (0.3 mM). As for kinetic parameters determination for NADH and NAD^+^, their concentrations were varied from 0.025 to 0.4 mM and from 0.1 to 20 mM, while the concentrations of other substrates were fixed at 40 mM (TMP) and 2 mM (L-leucine), respectively.

### Substrate Specificity

In view of the reversible reaction of oxidative deamination and reductive amination, different substrates were selected to analyze the substrate specificity of LeuDH. Oxidative deamination reaction was conducted in a 1.5 ml reaction system of glycine-NaOH buffer (100 mM, pH 9.5), including 5 mM different amino acids, 0.2 mM NAD^+^, and an appropriate amount of LeuDH enzyme solution incubated at 30°C for 2 min. The catalytic activity of LeuDH for L-leucine was defined as 100%, and the enzyme activity of LeuDH for other substrates was determined.

Ammonia reduction reaction was conducted in a 1.5 ml reaction system of NH_4_CI-NH_3_ H_2_O buffer (1 M, pH 8.5), including 5 mM different keto acids, 0.2 mM NADH, and an appropriate amount of LeuDH enzyme solution incubated at 30°C for 2 min. The catalytic activity of LeuDH for TMP was defined as 100%, and the enzyme activity of LeuDH for other substrates was determined.

### Enzymatic Production of L-*tert*-Leucine

In order to recycle the coenzyme NADH, two free recombinant LeuDH and FDH were coupled to construct the reaction system. The crude cell-free extracts prepared from the disruption of the recombinant cells by ultrasonication were used as the catalyst. The 20 ml reaction system (pH 8.5) contained ammonium formate (0.8 M), NAD^+^ (0.25 g), and a certain amount of cell-free extracts. The partial reaction solution was sampled at different time points to detect substrate conversion and product formation. The samples were treated immediately in a boiling water bath for 10 min to inactivate enzymes and then filtrated to remove the protein precipitates and monitored by high-performance liquid chromatography (HPLC).

### Analytical Methods

The substrate (TMP) was analyzed on a ZORBAX ECLIPSE AAA column (4.6 nm × 150 mm, 5 um), eluted with acetonitrile/0.01 mol⋅L^–1^ KH_2_PO_4_ in a volume ratio of 15:85 as mobile phase at a flow rate of 1.0 ml⋅min^–1^ and identified at 215 nm.

L-Tle was determined on the same column, while the mobile phase was composed of phase A (5 g⋅L^–1^ anhydrous sodium acetate, 200 μl triethylamine, 4 ml tetrahydrofuran solution, pH 7.2) and phase B (5 g⋅L^–1^ anhydrous sodium acetate, 400 ml methanol and acetonitrile, pH 7.2). The 20 min pump procedure was programmed as follows: the mobile phase A is reduced from 90 to 44% in 0–12 min, then decreased to 0% in 2.5 min, held for 2.5 min, and then increased to 90% in 3 min. Other control conditions include column temperature (40°C), injection volume (10 μl), the UV detection (338 nm), and flow rate (1.0 ml⋅min^–1^).

## Results and Discussion

### Enzyme Cloning, Expression, and Purification

In recent years, the genome mining strategy for the discovery of new enzyme genes, oriented by bioinformatics analysis, has attracted much attention. In the present study, genome mining was used to discover LeuDHs with high activities and other excellent performance *via* bioinformatics analysis based on the NCBI database. By using LeuDH with a high enzyme activity from *Bacillus sphaericus* (*Bsp*LeuDH) as templates (Li et al., 2009), alignment of amino acid sequences of LeuDH was conducted, wherein LeuDH genes from a wide range of microbes in the database were covered. In order to obtain LeuDH discrepant in the catalytic properties, LeuDHs with identity of 20–100% to *Bsp*LeuDH in amino acid sequences were selected from the NCBI database. Ten LeuDH genes were expressed in *E. coli* cells, and four LeuDHs presenting high activities were analyzed regarding with their thermostability. Results listed in Table 1 indicate that LeuDH from *E. sibiricum* (*Esi*LeuDH) shows the highest activity and half-life at 50°C. However, only 70% identity in amino acid sequences to *Bsp*LeuDH indicates that *Esi*LeuDH was quite different from *Bsp*LeuDH.

**TABLE 1:** Specific activity and thermostability of LeuDHs mined from NCBI.

**Origin**	**LeuDH**	**Identity (%)**	**Specific activity (U/mg protein)**	**Half-life at 50°C (h)**
*Exiguobacterium sibiricum*	*Esi*LeuDH	70.3	28.0	19.1
*Bacillus sphaericus*	*Bsp*LeuDH	100.0	27.4	14.4
*Lysinibacillus sphaericus*	*Lsp*LeuDH	95.1	15.7	11.0
*Bacillus cereus*	*Bce*LeuDH	79.1	23.9	10.0

*LeuDH, leucine dehydrogenase.*

**FIGURE 3:**
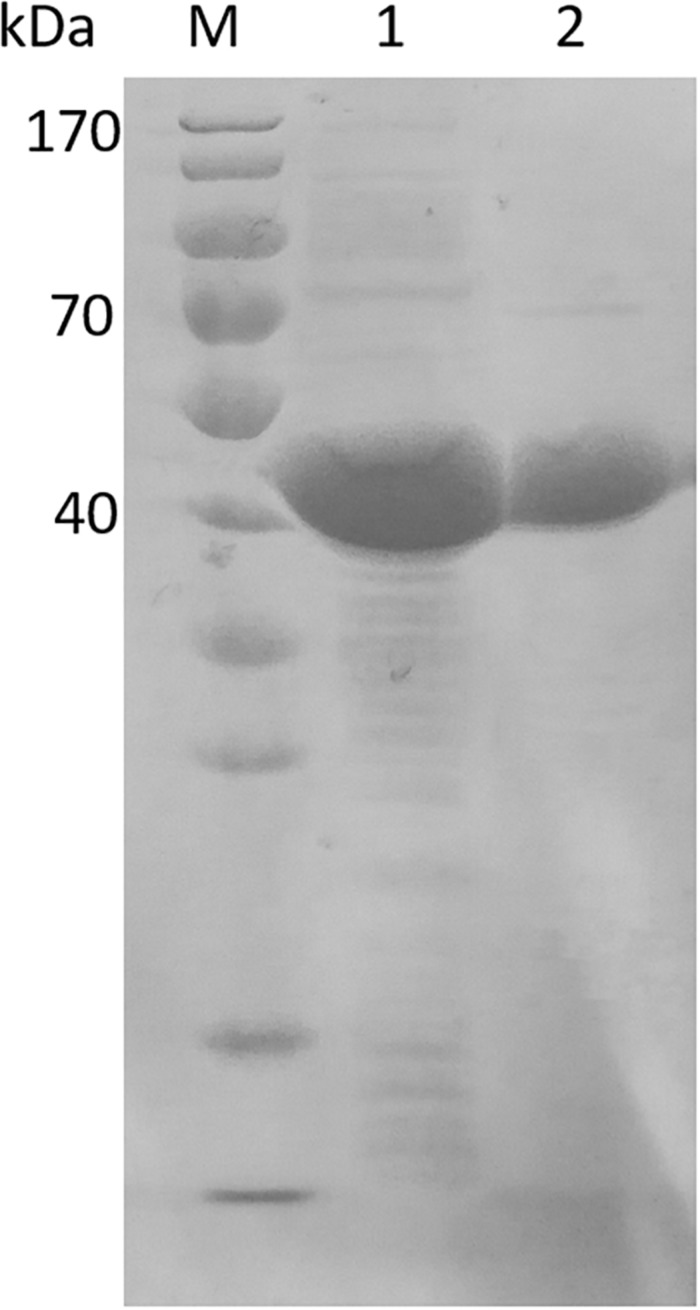
Electrophoresis of crude extract and purified *Esi*LeuDH. lane M, protein marker; lane 1, crude extract of *Esi*LeuDH; lane 2, purified *Esi*LeuDH.

In order to characterize *Esi*LeuDH, Ni^2+^-affinity chromatography was used to purify *Esi*LeuDH to homogeneity since the recombinant protein contains a 6-His tag. After one-step purification, the specific activity of the purified protein toward TMP was enhanced by 1.4-fold compared with that of the crude extract. SDS-PAGE analysis also demonstrates that the purification strategy removes almost all the impurity proteins, since the purified protein migrated as a single band (Figure 3). The molecular weight of *Esi*LeuDH is 40 kDa, coinciding with the theoretical value calculated according to its amino acid sequence.

### Optimum pH, Temperature, and Thermostability

**FIGURE 4:**
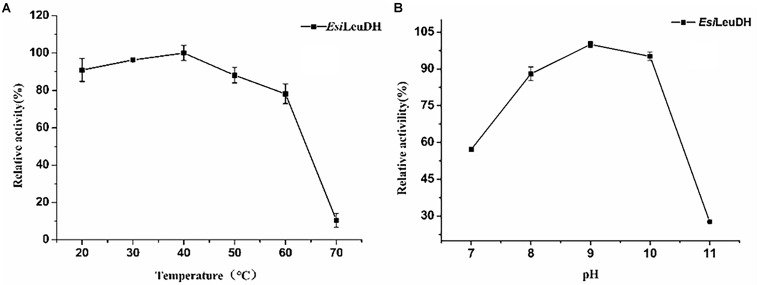
The optimum temperature and pH of the purified enzyme. **(A)** Optimum Temperature: the enzyme activity was measured at various temperatures (20–70°C) in 1 M NH_4_Cl-NH_3_⋅H_2_O buffer (pH 8.5). **(B)** Optimum pH: the enzyme activity was assayed at various pH (7.0–11.0) in NH_4_Cl-NH_3_⋅H_2_O buffer. Relative activity was expressed as a percentage of maximum activity under the experimental conditions.

In order to acquire the values for optimum pH and temperature, enzymatic activities of *Esi*LeuDH at various temperatures and pHs were measured. The optimum temperature of *Esi*LeuDH was 40°C, and the enzyme activity decreased rapidly when the temperature exceeded 60°C (Figure 4A). Jiang have shown that the optimum temperature of LeuDH from *Alcanivorax dieselolei* was 30°C (Zhu et al., 2016), but Zhu indicated that the value increased to 60°C when LeuDH from *Laceyella sacchari* was investigated (Jiang et al., 2016), suggesting significant differences in the catalystic properties of this enzyme from different species. Another important parameter for the purified *Esi*LeuDH is the optimal pH, which was determined as shown in Figure 4B. It can be seen that *Esi*LeuDH maintained a high enzyme activity under alkaline conditions (pH 8–10), which is much similar to those from *B. cereus* (optimum pH 9.0–9.5) (Schütte et al., 1985) and from *B. stearothermophilus* (optimum pH 8.8–9.7) (Ohshima et al., 1985a).

**FIGURE 5:**
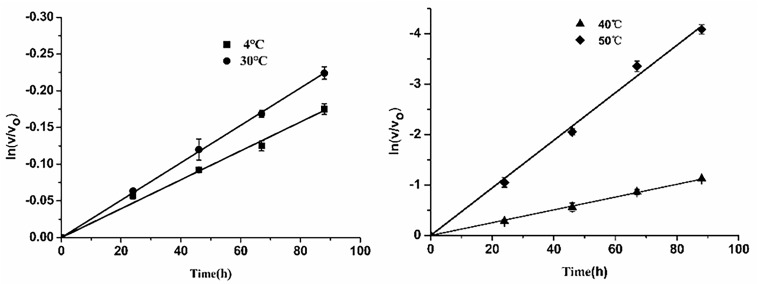
Thermostability of *Esi*LeuDH. The purified enzyme was pre-incubated in water at varied temperatures for a required period. Residual activity was expressed as a percentage of the activity measured at 0 h.

Subsequently, thermostability of *Esi*LeuDH was analyzed by measuring the reaction rate of *Esi*LeuDH under different temperatures. The half-lives of *Esi*LeuDH are 352 h (4°C), 272 h (30°C), 55 h (40°C), and 15 h (50°C), indicating that *Esi*LeuDH has a good thermostability (Figure 5).

### Influence of Metal Ions on the Activity of the Leucine Dehydrogenase Gene From *E. sibiricum*

In order to determine the effect of metal ions and ethylenediaminetetraacetic acid (EDTA) on the catalytic activity of *Esi*LeuDH, various kinds of metal ions or EDTA (1 mM) were supplemented into the enzyme solutions for 2-h incubation before the analyses of enzymatic activities (Table 2). None of them could activate the enzyme, while some ions (Na^+^, Ca^2+^, Mg^2+^, Mn^2+^, Cu^2+^, and Zn^2+^) present little effect on the enzyme activity of *Esi*LeuDH, and the relative residual enzyme activity was above 90.0% after 2 h at 30°C. However, Fe^2+^ and Fe^3+^ substantially reduced enzyme activity by 50%, indicating that *Esi*LeuDH does not require the abovementioned metals for its activity, similar to the reported LeuDH (Ye et al., 2015).

**TABLE 2:** Effect of metal ions and EDTA on *Esi*LeuDH activity.

**Metal ion (1 mM)**	**Relative activity (%)**
None	100 ± 2.7
Na^+^	102.74 ± 1.1
Ca^2+^	101.53 ± 3.8
Mg^2+^	97.04 ± 0.35
Mn^2+^	95.83 ± 5.5
Cu^2+^	95.29 ± 4.1
Zn^2+^	92.48 ± 1.7
Fe^2+^	59.5 ± 5.8
Fe^3+^	47.95 ± 3.9
EDTA	85.79 ± 1.7

**The EsiLeuDH was preincubated in 1 M NH_4_Cl-NH_3_⋅H_2_O buffer (pH 8.5) containing 1.0 mM of various metal ions or EDTA at 30°C for 2 h, then the activity toward TMP was measured under standard conditions. The control enzyme activity was assayed in the absence of any test compound.**

### Substrate Specificity and Enzyme Kinetics

LeuDH has the capacity to catalyze reactions in the opposite direction, i.e., reductive amination and oxidative deamination. Here, the substrate specificity of *Esi*LeuDH was investigated in terms of the above two types of biochemical reactions. As listed in Table 3, *Esi*LeuDH exhibits activities toward aliphatic α-keto acids. The enzyme activity for TMP was 15.7 U⋅ml^–1^, and for 2-oxobutyric acid was 39.3 U⋅ml^–1^. Meanwhile, *Esi*LeuDH also presents certain activity for the aromatic substrate such as phenylglyoxylic acid, similar to the reported LeuDHs (Li et al., 2014). *Esi*LeuDH also shows activity toward other aliphatic α-amino acids, especially the branched chain amino acids, which is similar to the observations for other known LeuDHs (Li et al., 2009).

**TABLE 3:** Substrate specificity of *Esi*LeuDH in reductive amination and oxidative deamination.

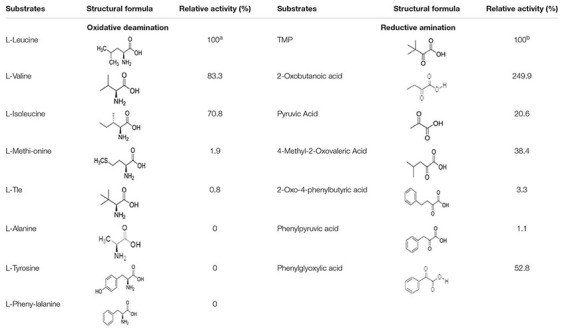

*^*a*^The initial rate of L-leucine measured was 10.5 U⋅ml^–1^, which was defined as 100% activity for oxidative deamination reaction. ^*b*^The initial rate of TMP measured was 15.74 U⋅ml^–1^, which was defined as 100% activity in the reductive amination reaction. *Esi*LeuDH, leucine dehydrogenase gene from Exiguobacterium sibiricum; TMP, trimethylpyruvic acid.*

Steady-state kinetics analysis of *Esi*LeuDH is another important task for the assessment of enzymatic properties, which is usually with the help of Lineweaver–Burk plot (Table 4). The *K*_m_ and *k*_cat_/*K*_m_ of *Esi*LeuDH for the substrate L-leucine were 0.88 mM and 4.90 L⋅mmol^–1^⋅s^–1^, respectively. The highest affinity indicates that L-leucine is the natural substrate of *Esi*LeuDH. The *K*_m_ and *k*_cat_/*K*_m_ of *Esi*LeuDH for TMP were 5.96 mM and 7.62 L⋅mmol^–1^⋅s^–1^, respectively, while the amino acid dehydrogenases from *E*. *siribicum* and *Bacillus clausii* have a *K*_m_ of 16.8 and 25.7 mM for TMP, respectively (Ye et al., 2015; Cheng et al., 2016). This comparison shows that *Esi*LeuDH has high affinity and catalytic level for TMP and can be used as a suitable catalyst for TMP conversion. In addition, *Esi*LeuDH has a large affinity for NAD^+^ and NADH (Table 4).

**TABLE 4:** Steady-state kinetic constants of *Esi*LeuDH.

**Substrate**	***K*_m_**	***V*_max_**	***k*_cat_**	***k*_cat_/*K*_m_**
	**(mM)**	**(μmol⋅min^–1^⋅mg^–1^)**	**(s^–1^)**	**(L⋅mmol^–1^⋅s^–1^)**
NADH	0.624	114.16	76.86	123.17
TMP	5.96	67.48	45.43	7.62
NAD	1.46	12.49	8.41	5.76
L-Leucine	0.88	6.4	4.31	4.90

*EsiLeuDH, leucine dehydrogenase gene from Exiguobacterium sibiricum; TMP, trimethylpyruvic acid.*

### Transformation of TMP to L-Tle by Recombinant Enzyme

#### Effect of pH on the Reaction

**FIGURE 6:**
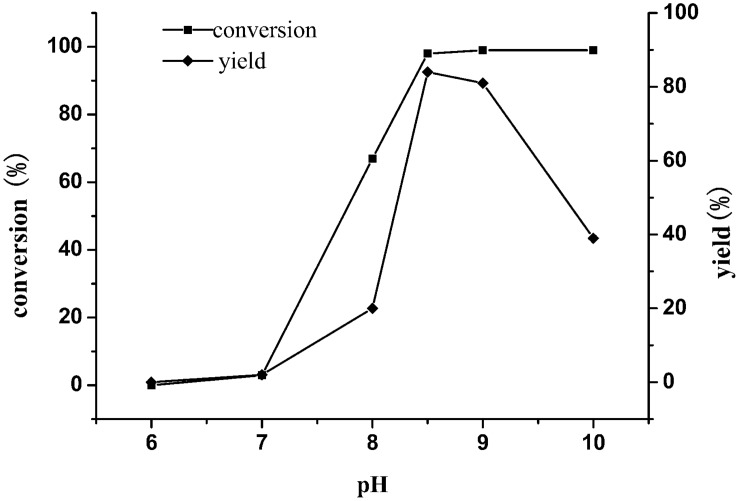
Effect of buffer pH on the reductive amination of TMP. Reaction conditions: TMP (0.3 M), ammonium formate (0.8 M), NAD^+^(0.25 g L^–1^) and cell-free extracts of *E. coli* cells (10 g L^–1^ *Esi*LeuDH, 20 g L^–1^ FDH) in PBS buffer (0.1 M), 30°C, 180 rpm, 12 h.

Previous studies have shown that the enzyme exhibited different catalytic activities under different pH conditions. In order to obtain good conversion of TMP, the enzymatic reactions were set under different pHs (6–10). As shown in Figure 6, incomplete conversion of substrate and low yield were seen under the condition of reaction pH less than 8.5. In contrast, the substrate can be completely converted, and the yield of the product was up to 85% at pH 8.5, indicating that pH 8.5 was the optimum value.

#### Effect of the Amount of Coenzyme and Auxiliary Substrate on the Reaction

In order to reduce the production cost of the reaction system, the additional amounts of the coenzyme NAD^+^ and the auxiliary substrate ammonium formate in the reaction were optimized. As shown in Table 5, the substrate TMP was hardly converted without the addition of coenzyme NAD^+^. The possible reason was that the enzyme activity of FDH was low during the catalytic process and insufficient NADH cannot be provided to provoke a reaction. Therefore, it is necessary to add some coenzyme and auxiliary substrate to promote the conversion of the substrate. The optimum additional amounts of NAD^+^ and ammonium formate were 0.25 g⋅L^–1^ and 0.8 M, respectively (Table 5).

**TABLE 5:** Effect of different amounts of coenzyme and auxiliary substrate on the reaction.

**NAD^+^ (g⋅L^–1^)**	**Ammonium formate (M)**	**Conversion (%)**	**Yield (%)**
0	0.8	8	2
0.1	0.8	34	27
0.2	0.8	81	33
0.25	0.8	99	70
0.25	0.4	70	48
0.25	0.6	84	53
0.25	0.8	99	71
0.25	1	99	74

#### Effect of Trimethylpyruvic Acid Content on the Reaction

In order to obtain L-Tle with a higher yield, the concentration of substrate TMP was tested. As the concentration of TMP increased, longer time was needed for the conversion of the substrate (Table 6). When the substrate concentration reached 0.6 M, the conversion rate of the reaction dropped to only 83%, indicating that the high concentration of TMP will produce feedback inhibition and cause incomplete conversion and low yield.

**TABLE 6:** Effect of different concentration of TMP on the reaction.

**TMP (M)**	***Esi*LeuDH**	**FDH**	**Time (h)**	**Conversion (%)**	**Yield (%)**
	**(g⋅L^–1^)**	**(g⋅L^–1^)**			
0.2	10	20	6	98	66
0.3	10	20	10	99	61
0.4	10	20	12	98	80
0.5	10	20	24	98	78
0.6	10	20	24	83	64
0.5	20	30	20	98	96
0.5	20	40	12	98	80

*EsiLeuDH, leucine dehydrogenase gene from Exiguobacterium sibiricum; FDH, formate dehydrogenase; TMP, trimethylpyruvic acid.*

#### Effect of Batch Addition of Trimethylpyruvic Acid on the Reaction

**FIGURE 7:**
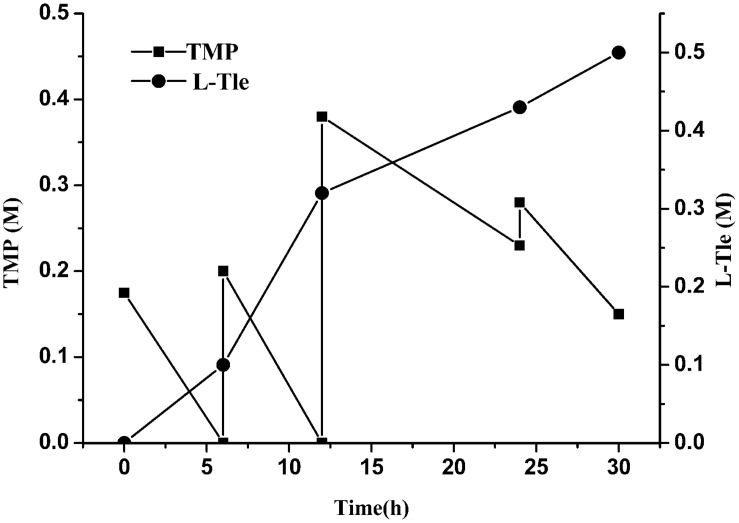
Effect of substrate feeding strategy on the reductive amination of TMP. Reaction conditions: four additions of TMP solution (1.5, 2, 4, 2.5 mL four times at 0, 6, 12, and 24 h, final concentration 0.8 M), ammonium formate (0.8 M), NAD^+^ (0.25 g L^–1^) and cell-free extracts of *E. coli* cells (10 g L^–1^ *Esi*LeuDH, 20 g L^–1^ FDH) in PBS buffer (0.1 M).

We observed that a high concentration of TMP could inhibit the reaction and reduce the yield of L-Tle. In order to reduce the substrate inhibition effect, the strategy of adding the substrate in batches was investigated. As shown in Figure 7, TMP was supplemented into the reaction solution in batches to sustain a low concentration (no more than 40 mM). TMP added in the first two rounds can be completely converted into product, while the additional supplement of TMP causes some remaining of TMP. The total concentration of L-Tle was 0.5 M (65.6 g⋅L^–1^) with an average conversion rate of 81% after 30 h of reaction. The strategy of batch addition of TMP alleviated the inhibition of the substrate to some extent and increased the yield of the product.

## Conclusion

By means of genome mining strategy, the LeuDH gene from *E*. *sibiricum* was isolated and identified. Gene cloning, expression, and purification enabled us to obtain the purified protein of this enzyme, which has excellent enzymatic properties in research or industrial application. *Esi*LeuDH is much stable, with a half-life of 14.7 h at 50°C. In addition, *Esi*LeuDH shows a broad spectrum of substrates and exhibits good catalytic activity for most aliphatic amino acids, keto acids, and some aromatic keto acids (e.g., phenylglyoxylic acid).

The synthesis of L-Tle through conversion of TMP was investigated by using a coupling system containing cell-free extracts of LeuDH and FDH recombinant strains. After optimization, 0.8 M of TMP was converted to L-Tle under fed-batch mode. Finally, an average conversion rate of 81% was achieved, and the concentration of L-Tle was 65.6 g⋅L^–1^. This study lays the foundation for the synthesis of chiral non-natural amino acids by using *Esi*LeuDH.

## Data Availability Statement

All datasets generated for this study are included in the article/supplementary material.

## Author Contributions

WL and ZR designed the experiments. JZ, YZ, and HZ performed the discovery, cloning, and expression and analyses of the novel enzymes, while XuY, YL, and XiY performed the L-*tert*-leucine production. WL and JZ wrote the manuscript and revised it until it was accepted.

## Conflict of Interest

XuY and YL were employed by Hulunbeier Northeast Fufeng Biotechnologies Co., Ltd.

The remaining authors declare that the research was conducted in the absence of any commercial or financial relationships that could be construed as a potential conflict of interest.
